# Statin use and the prognosis of patients with hepatocellular carcinoma: a meta-analysis

**DOI:** 10.1042/BSR20200232

**Published:** 2020-04-03

**Authors:** Xiaofei Li, Liwen Liu, Yongtao Hu

**Affiliations:** Department of Infectious Diseases, Yiwu Central Hospital, Yiwu 322000, China

**Keywords:** Statin, Hepatocellular carcinoma, Mortality, Recurrence, Meta-analysis

## Abstract

**Background:** Association between statin use and prognosis in patients with hepatocellular carcinoma (HCC) remains unknown. We performed a meta-analysis of follow-up studies to systematically evaluate the influence of statin use on clinical outcome in HCC patients.

**Methods:** Studies were obtained via systematic search of PubMed, Cochrane’s Library, and Embase databases. A randomized-effect model was used to pool the results. Subgroup analyses were performed to evaluate the influence of study characteristics on the association.

**Results:** Nine retrospective cohort studies were included. Overall, statin use was associated with a reduced all-cause mortality in HCC patients (risk ratio [RR]: 0.81, 95% CI: 0.74–0.88, *P* < 0.001; *I*^2^ = 63%). Subgroup analyses showed similar results for patients with stage I-III HCC (RR: 0.83, 0.79, and 0.90 respectively, *P* all < 0.01) and patients after palliative therapy for HCC (RR: 0.80, *P* < 0.001), but not for patents with stage IV HCC (RR: 0.91, *P* = 0.28) or those after curative therapy (RR: 0.92, *P* = 0.20). However, the different between subgroups were not significant (both *P* > 0.05). Moreover, statin use was associated with reduced HCC-related mortality (RR: 0.78, *P* = 0.001) in overall patient population and HCC recurrence in patients after curative therapies (RR: 0.55, *P* < 0.001).

**Conclusions:** Satin use is associated with reduced mortality and recurrence of HCC. These results should be validated in prospective cohort studies and randomized controlled trials.

## Introduction

Hepatocellular carcinoma (HCC) is a prevalent cancer in digestive system, and it has been estimated that over 500,000 patients are newly diagnosed as HCC each year all over the world [1]. Currently, the treatment strategies for patients with HCC are limited, and patients with HCC are of poor prognosis, with a reported median survival of <1 year [2–4]. Statins, also known as 3-hydroxy-3-methylglutaryl CoA (HMG-CoA) reductase inhibitors, are a category of cholesterol-lowering medications that have become mainstays for the primary and secondary prevention of cardiovascular diseases [5]. Moreover, the potential pleiotropy of statins besides their cholesterol-lowering effect has been revealed, such as anti-inflammation, immunomodulation, pro-apoptosis, anti-proliferation, and anti-invasion, all of which are implicated in carcinogenesis and metastasis [6,7]. Accordingly, statins have been proposed to confer anticancer effect, and use of statin may reduce cancer risk [8]. A previous meta-analysis suggested that compared with people who never take satins, those who take statins have a 37% reduced HCC incidence [9]. Moreover, a recent meta-analysis showed that treatment of statin improved portal vessel pressure and clinical outcomes in patients with cirrhosis, including a lowered occurrence of HCC [10]. These findings demonstrated that statin use is associated with reduced risk of HCC. However, in patients with diagnosed HCC, whether post-diagnosis statin use could improve their clinical outcomes remains unknown. Pilot studies evaluating the potential influence of statin use on clinical outcomes in patients with HCC retrieved inconsistent results. Although some studies suggested that statin use may be related with improved survival in HCC patients [11–13], other studies did not [14–17]. Therefore, in the present study, we aimed to perform a meta-analysis of follow-up studies to systematically evaluate the association between statin use and clinical outcomes in HCC patients.

## Methods

The meta-analysis was performed in accordance with the MOOSE (Meta-analysis of Observational Studies in Epidemiology) [18] and Cochrane’s Handbook [19] guidelines.

### Literature search

Studies were identified via systematic search of electronic databases of PubMed Cochrane’s Library, and Embase via the following terms: (1) ‘statin’ OR ‘3-hydroxy-3-methyl-glutaryl CoA reductase inhibitor’ OR ‘CS-514’ OR ‘statin’ OR ‘simvastatin’ OR ‘atorvastatin’ OR ‘fluvastatin’ OR ‘lovastatin’ OR ‘rosuvastatin’ OR ‘pravastatin’ OR ‘pitavastatin’; and (2) ‘hepatocellular cancer’ OR ‘hepatocellular tumor’ OR ‘hepatocellular carcinoma’ OR ‘hepatocellular neoplasm’ OR ‘liver cancer’ OR ‘liver tumor’ OR ‘liver carcinoma’ OR ‘liver neoplasm’ OR ‘HCC’. We applied this extensive search strategy to avoid potentially missing of available studies. The search was limited to human studies without language restriction. The reference lists of related original and review articles were also analyzed using a manual approach. The final literature search was performed on November 22, 2019.

### Study selection

The inclusion criteria for the studies were: (1) longitudinal follow-up studies published as full-length articles, including randomized controlled trials (RCTs), cohort studies, and nested case–control studies; (2) included at least 100 adult patients with HCC at baseline; (3) evaluated the association between statin use and at least one of the following outcomes, including all-cause mortality, HCC-related mortality, and tumor recurrence; (4) with a minimal follow-up duration of 1 year; and (5) reported the relative risk for this association after adjustment of potential confounding factors. Reviews, editorials, preclinical studies, and studies irrelevant to the aim of current meta-analysis were excluded.

### Data extracting and quality evaluation

Literature search, data extraction, and quality assessment of the included studies were independently performed by two authors according to the predefined criteria. Discrepancies were resolved by consensus or discussion with the corresponding author. The extracted data included: (1) name of first author, publication year, and country where the study was performed; (2) study design characteristics; (3) patient characteristics, including sample size, age, sex, prevalence of hepatitis B or C virus (HBV or HCV) infection, TNM stages of the tumor, and proportions of patients that received curative therapies for HCC, including hepatic resection, liver transplantation, radiofrequency ablation, and percutaneous ethanol injection [20]; (4) definition of statin use; (5) follow-up durations; (6) outcomes reported; and (7) confounding factors adjusted when presenting the association between statin use and clinical outcomes in patients with HCC. The quality of each study was evaluated using the Newcastle–Ottawa Scale [21] that ranges from 1 to 9 stars and judges each study regarding three aspects: selection of the study groups; the comparability of the groups; and the ascertainment of the outcome of interest.

### Statistical analyses

We used risk ratios (RRs) and their corresponding 95% confidence intervals (CIs) as the general measure for the association between statin use and clinical outcomes. Data of RRs and their corresponding stand errors (SEs) were calculated from 95% CIs or *P* values, and were logarithmically transformed to stabilize variance and normalized the distribution [19]. The Cochrane’s *Q* test and estimation of *I*^2^ statistic were used to evaluate the heterogeneity among the include cohort studies [22]. A significant heterogeneity was considered if *I*^2^ > 50%. We used a randomized-effect model to synthesize the RR data because this model is considered as a more generalized method that incorporates the potential heterogeneity among the included studies [19]. Sensitivity analyses, by omitting one individual study at a time, were performed to test the robustness of the results [23]. Predefined subgroup analyses were performed to evaluate the influences of study characteristics on the main outcome of all-cause mortality, such as HBV infection, TNM stages, and patients that received curative or palliative therapies. The potential publication bias was assessed by funnel plots with the Egger’s regression asymmetry test [24]. If publication bias was detected, we used the ‘trim-and-fill’ analyses to evaluate the potential influence of imputed unpublished studies with negative results on the outcome [19]. This method incorporated the hypothesized unpublished studies to generate symmetrical forest plots. We used the RevMan (Version 5.1; Cochrane Collaboration, Oxford, U.K.) and STATA software for the meta-analysis and statistics.

## Results

### Literature search

**Figure 1:**
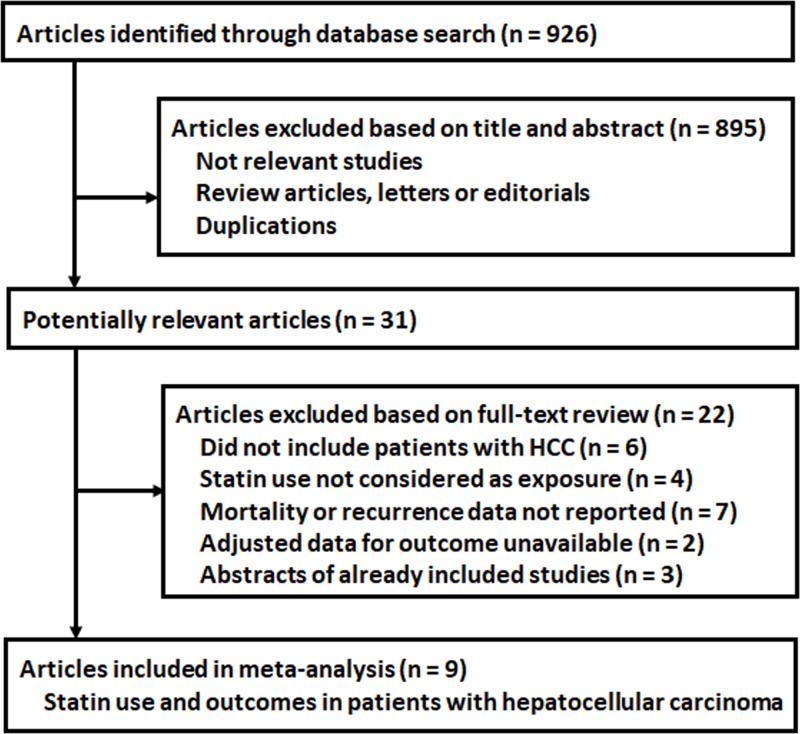
Flow chart of database search and study identification

The process of database search was summarized in Figure 1. Briefly, 926 articles were found via initial literature search of the PubMed, Cochrane’s Library, and Embase databases, and 895 were excluded through screening of the titles and abstracts mainly because they were not relevant to the purpose of the meta-analysis. Subsequently, 31 potential relevant records underwent full-text review. Of these, 22 were further excluded because six studies did not include HCC patients, four studies did not valuate statin use as exposure, seven studies did not evaluate mortality or recurrence outcomes in HCC patients, two studies did not contain available data for the multivariate adjusted association between statin use and clinical outcomes in HCC patients, and the remaining three were abstracts of already included studies. Finally, nine studies were included [11–17,25,26].

### Study characteristics and quality evaluation

The characteristics of the included studies are summarized in Table 1. All of the included studies were retrospective cohort studies. Since two studies reported data according to HBV infection status of the patients [11,12], and one of them also reported stratified data according to the TNM stages of the tumor [11], these datasets were included separately. Overall, nine retrospective cohort studies including 62,273 HCC patients from China [11,12,16,25], Japan [14,15], Korea [26], and the United States [13,17] were included. The mean age of the included patients varied from 54 to 67 years, with proportions of male ranging from 64% to 99%. Five studies included patients after curative treatments [14–16,25,26], two studies included patients with palliative treatments [11,12], while the remaining two studies included patients with either treatments [13,17]. Statin use was validated by prescription records in all of the included studies and defined by accumulative statin dose of ≥28 to 30 cumulative defined daily dose (cDDD) in most studies [11,12,14–16,26]. The follow-up duration varied from 1.7 to 4.4 years. Potential confounding factors, such as age, sex, comorbidities, tumor stages, anticancer treatments, and other concurrent medications, were adjusted when presenting the outcome in all of the included studies. The NOS scores of the included studies ranged from six to eight, indicating generally good study quality.

**Table 1:** Characteristics of the included follow-up studies

Study	Design	Country	Patient number	Mean age	Male	HBV infection	HCV infection	TNM stage	Curative therapy	Definition of statin use	Follow-up durations	Outcomes reported	Variables adjusted	NOS
				Years	%	%	%		%		Years			
Wu, 2012	RC	China	4569	54.6	82.3	100	0	NR	100	Statin use of >1 tablet/month during follow-up	2.2	HCC recurrence	Age, sex, resection extent, liver cirrhosis, DM, and use of NSAIDs, aspirin, and metformin	8
Shao, 2015	RC	China	20200	59.8	80.2	NR	NR	I-IV	0	Any statin use >28 cDDD during follow-up	1.7	HCC related mortality and all-cause mortality	Age, sex, nucleoside/nucleotide analogs, interferons, aspirin, metformin, cirrhosis, liver failure, HTN, and DM	8
Jeon, 2016	RC	the US	1036	NR	64.3	11.3	29.6	I-II	36.5	Any statin use during follow-up	1.8	All-cause mortality	Age, sex, race, income, tumor stages, tumor treatment, cirrhosis, HTN, and DM	7
Wu, 2016	RC	China	18892	64.3	70.4	54.9	NR	I-IV	0	Any statin use >28 cDDD during follow-up	1.7	HCC related mortality and all-cause mortality	Age, sex, tumor stage, nucleoside/nucleotide analogues, interferons, aspirin, metformin, cirrhosis, liver failure, HTN, DM, COPD, ACS, and stroke	8
Kawaguchi, 2017	RC	Japan	734	67.1	77.2	22.1	43.9	NR	100	Any statin use >28 cDDD during follow-up	3.1	HCC recurrence and all-cause mortality	Age, sex, tumor size, vascular invasion, liver cirrhosis, HBV or HCV infection, and surgical characteristics	6
Nishio, 2018	RC	Japan	643	66.7	77.9	NR	NR	NR	100	Any statin use >28 cDDD during follow-up	NR	HCC recurrence and all-cause mortality	Age, sex, BMI, Child-Pugh Class, AFP, tumor size, vascular invasion, liver cirrhosis, HTN, and DM	6
Thrift, 2019	RC	the US	15422	63.1	99.2	9.3	68.4	I-IV	24.7	Any statin use during follow-up	NR	HCC related mortality and all-cause mortality	Age, sex, race, BMI, alcohol abuse, smoking status, HBV or HCV infection, cirrhosis, tumor stage, MELD score, tumor treatment, and use of NSAIDs, aspirin, and metformin	8
Young, 2019	RC	China	430	58.4	85.1	100	5.4	NR	100	Any statin use >30 cDDD during follow-up	4.2	HCC recurrence and all-cause mortality	Age, sex, smoking, cirrhosis, tumor stage, DM, HTN, and use of NSAIDs, aspirin, and metformin	6
Cho, 2019	RC	Korea	347	55.3	82.4	81.8	8.6	NR	100	Any statin use >30 cDDD during follow-up	4.4	HCC recurrence	Age, sex, tumor stage, vascular invasion, liver cirrhosis, and AFP	6

Abbreviations: ACS, acute coronary syndrome; AFP, alpha-fetoprotein; BMI, body mass index; cDDD, cumulative defined daily dose; COPD, chronic obstructive pulmonary disease; DM, diabetes mellitus; HBV, hepatitis B virus; HCV, hepatitis C virus; HCC, hepatocellular carcinoma; HTN, hypertension; MELD, Model for End-stage Liver Disease; NOS, the Newcastle–Ottawa Scale; NR, not reported; NSAID, nonsteroidal anti-inflammatory drug; RC, retrospective cohort; US, United States.

### Statin use and all-cause mortality in HCC patients

**Figure 2:**
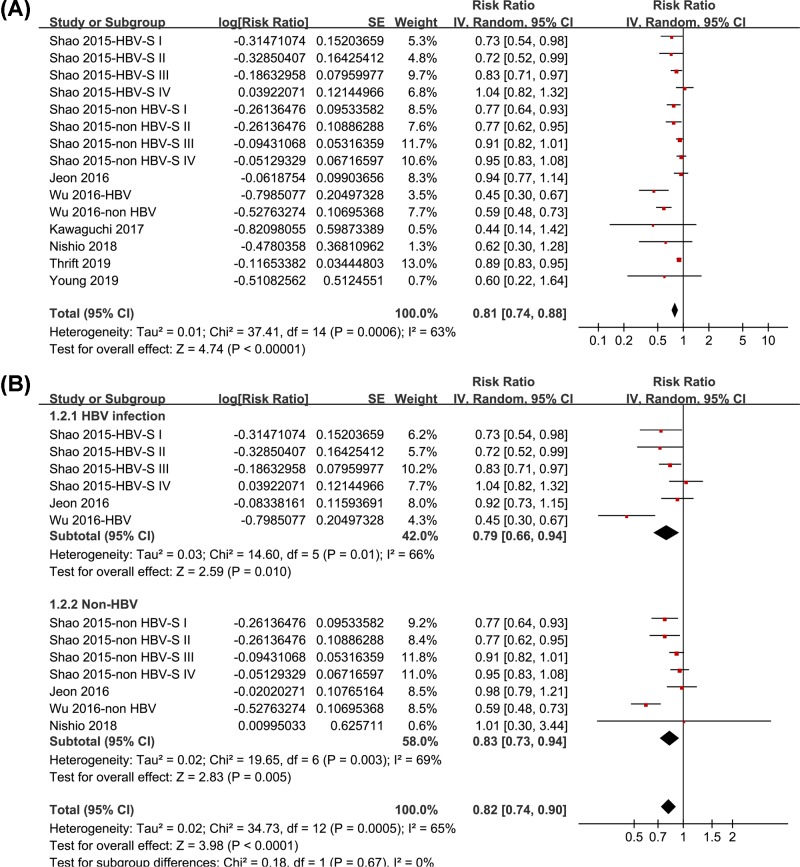
Forest plots for the meta-analysis of the association between statin use and all-cause mortality in HCC patients (**A**) Overall meta-analysis and (**B**) subgroup analyses according to HBV infection status.

**Figure 3:**
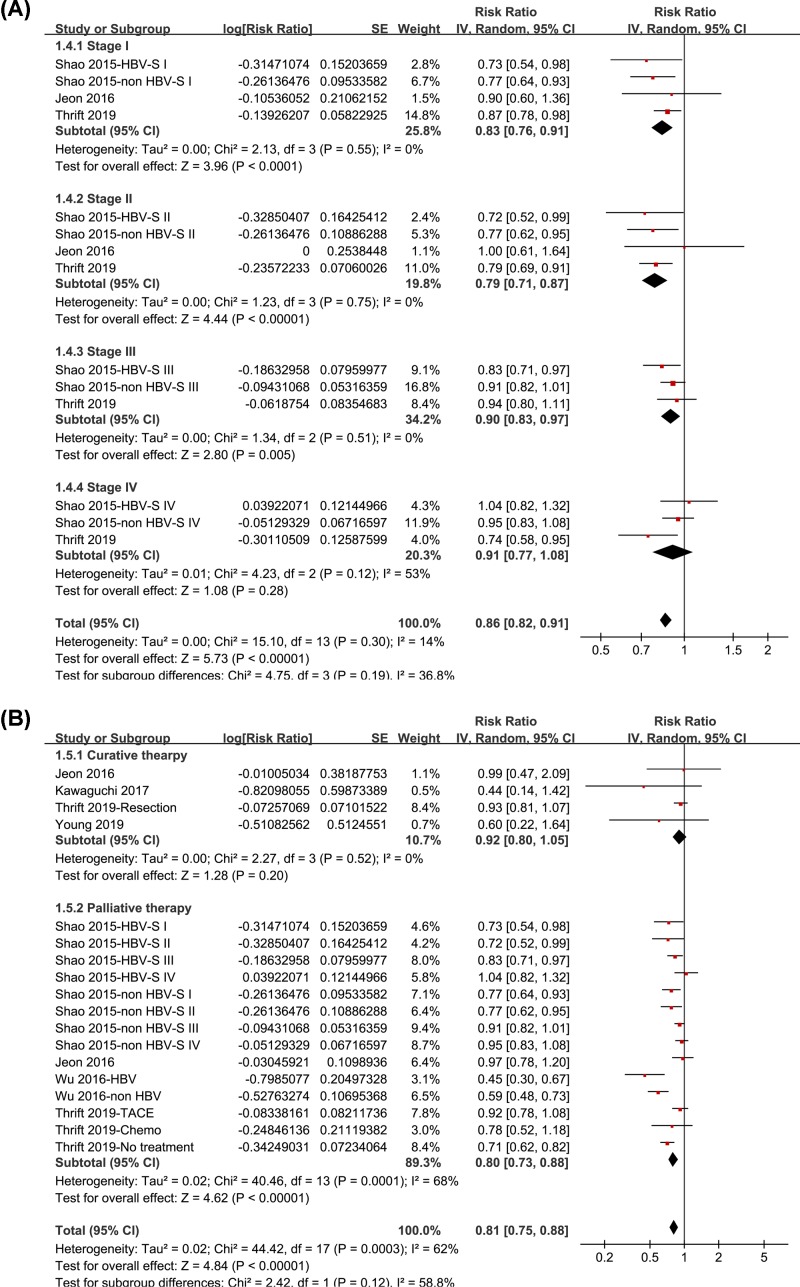
Subgroup analyses for the meta-analysis of the association between statin use and all-cause mortality in HCC patients (**A**) Subgroup analyses according to the tumor stages and (**B**) subgroup analyses according to the treatments of the patients.

Pooled results of 15 datasets from 7 studies [11–17] using a randomized-effect model showed that statin use was associated with a reduced risk of all-cause mortality in patients with HCC (RR: 0.81, 95% CI: 0.74–0.88, *P* < 0.001; Figure 2A) with significant heterogeneity (*P* for Cochrane’s *Q* test <0.001, *I*^2^ = 63%). Sensitivity analyses by omitting one datasets at a time did not significantly change the results (RR: 0.79–0.84, *P* all <0.05). Subgroup analyses showed that statin use was associated with reduced risk of all-cause mortality in HCC patients with (RR: 0.79, 95% CI: 0.66–0.94, *I*^2^ = 66%, *P* = 0.01) and without HBV infection (RR: 0.83, 95% CI: 0.73–0.94, *I*^2^ = 69%, *P* = 0.005; *P* for subgroup difference = 0.67; Figure 2B). Moreover, subgroup analyses also showed that statin use was associated with reduced mortality in patients with stage I-III HCC (RR: 0.83, 0.79, and 0.90, respectively; *P* all <0.01; Figure 3A) and patients after palliative therapy for HCC (RR: 0.80, *P* < 0.001; Figure 3B), but not for patents with stage IV HCC (RR: 0.91, *P* = 0.28; Figure 3A) or those after curative therapy (RR: 0.92, *P* = 0.20; Figure 3B). However, the differences between the subgroups were not significant (both *P* > 0.05).

### Statin use and HCC-related mortality and HCC recurrence

**Figure 4:**
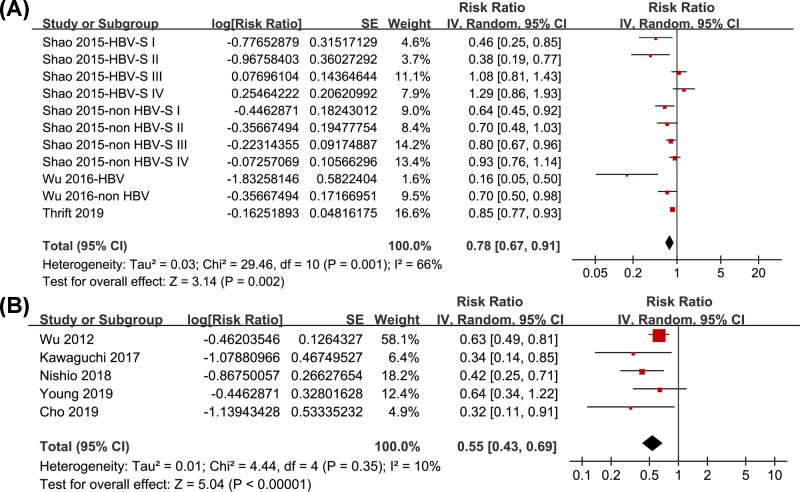
Forest plots for the meta-analysis of the association between statin use and the outcomes HCC-related mortality and HCC recurrence (**A**) HCC-related mortality; and (**B**) HCC recurrence.

Pooled results of 11 datasets from three studies [11–13] showed that statin use was associated with a reduced risk of HCC-related mortality (RR: 0.78, 95% CI: 0.67–0.91, *I*^2^ = 66%; *P* = 0.002; Figure 4A). Sensitivity analyses by omitting one datasets at a time did not significantly change the results (RR: 0.75–0.81, *P* all < 0.05). Pooled results of five studies [14–16,25,26] showed that statin use was associated with a reduced HCC recurrence in patients after curative therapy (RR: 0.55, 95% CI: 0.43–0.69, *P* < 0.001; Figure 4B). Sensitivity analyses by omitting one datasets at a time showed consistent results (RR: 0.45–0.59, *P* all < 0.05).

### Publication bias

**Figure 5:**
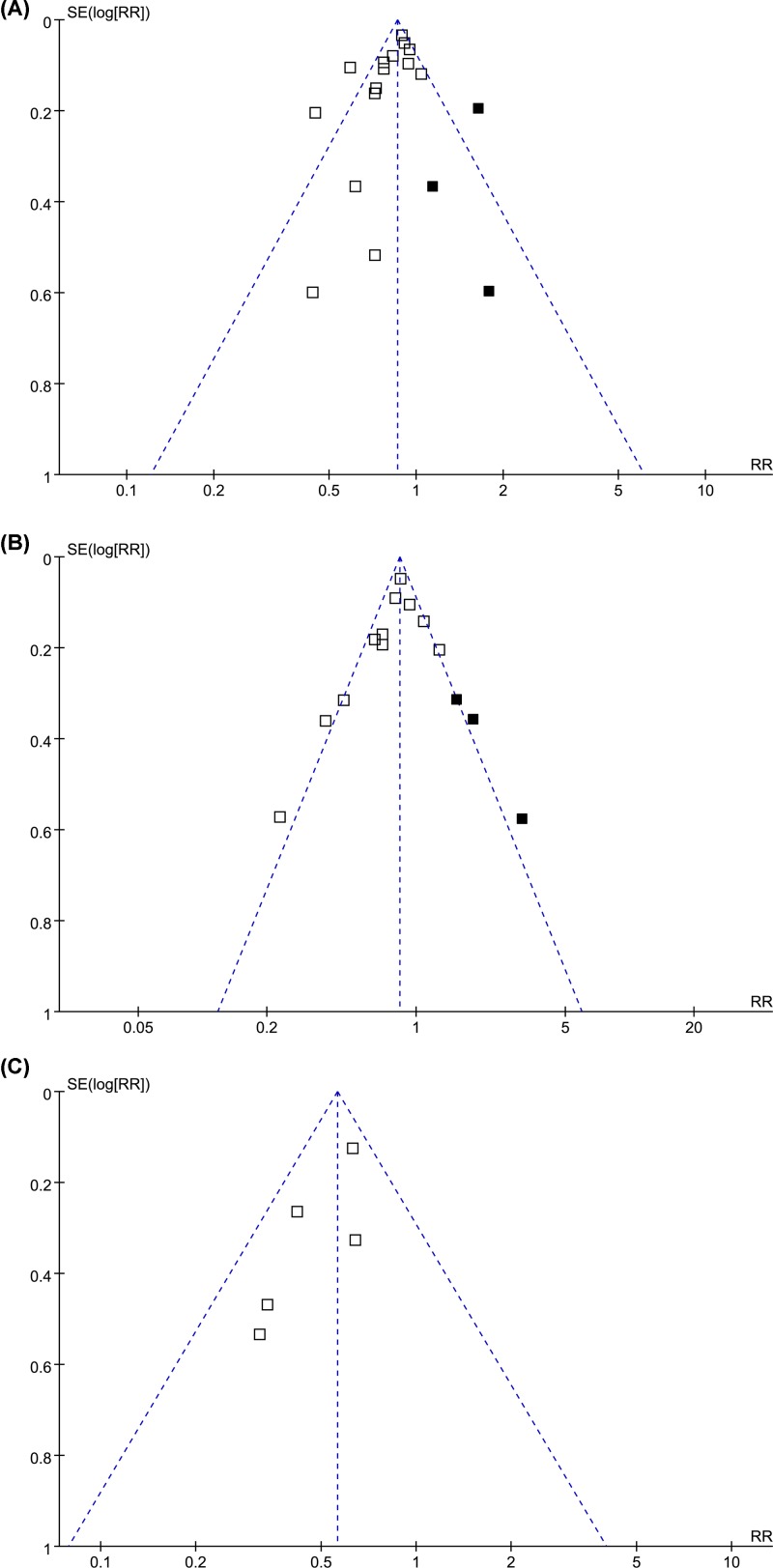
Funnel plots for the publication bias underlying the meta-analysis (**A**) Funnel plots with ‘trim-and-fill’ analyses for the meta-analysis between statin use and all-cause mortality in HCC patients. (**B**) Funnel plots with ‘trim-and-fill’ analyses for the meta-analysis between statin use and HCC-related mortality; and (**C**) funnel plots for the meta-analysis between statin use and HCC recurrence; the black squares indicate imputed studies with negative findings to generate symmetrical funnel plots

The funnel plots for the outcomes of all-cause mortality and HCC-related mortality were asymmetrical on visual inspection, suggesting potential risks of publication biases, which were consistent with the results of Egger’s regression tests (*P* = 0.032 and 0.048, respectively; Figure 5A,B). We used ‘trim-and-fill’ analyses to generate symmetrical funnel plots via incorporating hypothesized studies with negative results, and meta-analyses by including these studies did not significantly affect the results (all-cause mortality: RR = 0.84, 95% CI: 0.76–0.92, *P* < 0.001; and HCC-related mortality: RR = 0.83, 95% CI: 0.71–0.97, *P* = 0.02). The funnel plots for the meta-analysis between statin use and HCC recurrence were symmetrical on visual inspection (Figure 5C), indicating low risk of publication bias. Egger’s regression test was not performed since only five datasets were included.

## Discussion

By summarizing the current evidence from cohort studies, our meta-analysis showed that statin use is independently associated with reduced risk of all-cause mortality in HCC patients. Moreover, subgroup analyses showed that statin used was associated with reduced mortality risk in patients with or without HBV infection, in patients with TNM stage I-III HCC, and in HCC patients that received palliative treatments. In addition, statin use is associated with reduced HCC-related mortality and HCC recurrence. Taken together, these results demonstrated that statin use is independently associated with a reduced mortality risk of in HCC patients. Although large-scale prospective cohort studies and RCTs are needed to validate these findings, results of this meta-analysis support the potential role of statins as chemoprevention agents in HCC patients.

To the best of our knowledge, our study is the first meta-analysis focusing on the association between statin use and prognosis in HCC patients. The strengths of our study included follows. First, this meta-analysis included only longitudinal follow-up studies, which could therefore establish a sequential association between statin use and improved survival in HCC patients. Second, we only studies with adequate adjustment of confounding factors, which therefore may suggest an independently association between statin use and improved clinical outcomes in these patients. Third, we used sensitivity analysis to confirm the robustness of the finding, which showed that the results were stable and not primarily driven by either of the included study. Finally, multiple subgroup analyses were performed to evaluate the stability of the results, which showed that association between statin use and reduced overall mortality was consistent in patients with or without HBV infection, in patients with TNM stage I-III HCC, and in HCC patients that received palliative treatments. A previous meta-analysis confirmed that statin use is associated with reduced risk of HCC in overall population [9]. Our results further expanded these findings by showing that statins may confer anticancer effect in HCC patients, and use of statins may be associated with improved clinical outcomes in these patients. In view of the limited therapeutic options and poor prognosis of HCC patients in current clinical practice, large-scale prospective studies and RCTs are needed to validate the potential benefits of statins on clinical outcomes in these patients.

Results of our subgroup analyses showed that statin use was associated with reduced all-cause mortality in patients with stage I-III HCC but not in patients with stage IV HCC, and in patients after palliative treatments but not in those after curative treatments. However, the differences between the subgroups were not statistically significant, which indicates that the non-significant findings in subgroups of patients with stage IV HCC or patients after curative treatments could be due to the limited datasets included. Indeed, three and four datasets were available respectively for these two subgroups. In fact, patients with early stage of HCC are more likely to receive curative therapies, and the appearing controversial findings for patients with stage I-III HCC and those after curative therapies may reflect the subgroup results could be caused by limited datasets. Association between statin use and mortality risk in patients with stage IV HCC or patients after curative treatments should be investigated in future studies. When performing the database search, one potentially related RCT was noticed [27]. This pilot study published in 2001 included 83 advanced HCC patients showed that patients randomized to a daily dose of 40 mg pravastatin was associated with a longer median survival (18 months) than controls (9 months). The present study was excluded from our meta-analysis since they did not report adjusted RR for mortality. Collectively, benefits of statins on clinical prognosis of HCC patients should be validated in large-scale RCTs in the future.

Experimental studies have revealed some potential mechanisms underlying the chemoprevention effects of statins for HCC. Combinatorial treatment with statin and protein kinase C-beta inhibitor was shown to display enhanced anti-tumor efficacy in cultured HCC cells and in a mouse model of HCC [28]. Moreover, inhibition of HMG-CoA reductase by atorvastatin was accompanied with blockages of both MYC phosphorylation and activation, suppressed tumor initiation and growth in vivo in a transgenic model of MYC-induced HCC as well as in human HCC-derived cell lines [29]. Besides, treatment with fluvastatin, simvastatin, atorvastatin, rosuvastatin, or lovastatin were all associated with induced cellular apoptosis in mouse and human HCC cell lines in a p53 dependent manner [30]. In addition, other molecular pathways, such as inhibition of signal transducer and activator of transcription 3/SKP2 axis [31], inhibition of SRC/FAK cue [32], and activation of AMPK et al. [33] have also been involved in the potential anti-HCC effects of statins. Besides, many other benefits of statins in patients with hepatic disorders, such as cirrhosis, have also been noticed. In a recently published meta-analysis, statin use was associated with lowered hepatic portal vessel pressure, reduced HCC incidence, and improved survival in patients with liver cirrhosis, without significant influence on the risk of esophageal variceal bleeding or spontaneous bacterial peritonitis [10]. Future studies are warranted to uncover the key mechanisms underlying the potential anti-HCC efficacy and other benefits of statins in patients with hepatic diseases.

Our study has limitations that should be noticed when interpreting the results. First, significant heterogeneity exists among the included studies. Although subgroup analyses were performed to evaluate HBV status, tumor stages, and treatment options on the association between statin use and prognosis in HCC patients, we could not exclude that other study characteristics may also contribute to the heterogeneity, such as concurrent medications including antiviral agents [34] and use of metformin [35], which have both been indicated to confer anticancer effects. Furthermore, as mentioned above, due to the limited datasets available, results of subgroup analyses for the association between statin use and overall mortality in stage IV HCC patients and those received curative therapies should be cautious interpreted. Second, we were unable to determine whether the association between statin use and improved survival in HCC patients were independent of dose, treatment duration, and characteristics of the individual statin used (lipophilic or hydrophilic) because these data were rarely analyzed in the included studies. Third, our meta-analyses were based on study-level data rather than individual-patient level data, which also limited the reliability of stratified analyses. In addition, significant publication biases were noticed for meta-analysis of the associations between statin use and all-cause or HCC-related mortality in HCC patients. Although we applied ‘trim-and-fill’ analyses to show that incorporating the imputed studies with negative findings did not significantly change the results, publication biases may confound the significance of our findings. Moreover, although we included studies with adjusted data, we could not exclude the existence of residual factors which may confound the association. Finally, a causative association between statin use and reduced mortality risk in HCC patients could not be derived based on our finding since the present study was a meta-analysis of observational studies. Our findings should be treated as hypothesis-generating, and large-scale prospective studies and RCTs are needed to validate the potential benefits of statins on clinical outcomes in these patients.

In conclusion, results of meta-analysis demonstrated that statin use may be associated with improved survival and reduced recurrence in HCC patients. The chemoprevention efficacy of statin on clinical outcomes in HCC patients should be validated in large-scale prospective studies and RCTs.

## Abbreviations

ACS: acute coronary syndrome
AFP: alpha-fetoprotein
BMI: body mass index
cDDD: cumulative defined daily dose
COPD: chronic obstructive pulmonary disease
DM: diabetes mellitus
HBV: hepatitis B virus
HCC: hepatocellular carcinoma
HCV: hepatitis C virus
HTN: hypertension
MELD: Model for End-stage Liver Disease
NOS: the Newcastle–Ottawa Scale
NSAID: nonsteroidal anti-inflammatory drug
